# Humanitarian paediatrics: A statement of purpose

**DOI:** 10.1371/journal.pgph.0001431

**Published:** 2022-12-30

**Authors:** Daniel Martinez Garcia, Ribka Amsalu, Christian Harkensee, Sophie Janet, Ayesha Kadir, Vinay N. Kampalath, Sahar Nejat, Paul H. Wise

**Affiliations:** 1 Geneva University Paediatric Hospital, Geneva, Switzerland; 2 Médecins Sans Frontières, Toronto, Canada; 3 California Preterm Birth Initiative, Department of Obstetrics, Gynaecology, & Reproductive Sciences, University of California San Francisco, San Francisco, San Francisco, California, United States of America; 4 Queen Elizabeth Hospital, Gateshead, United Kingdom; 5 Médecins Sans Frontières, Dakar, Senegal; 6 Save the Children, London, United Kingdom; 7 Division of Emergency Medicine, Department of Paediatrics, Children’s Hospital of Philadelphia and University of Pennsylvania School of Medicine, Philadelphia, Pennsylvania, United States of America; 8 Independent Researcher, Stockholm, Sweden; 9 Stanford University School of Medicine, Stanford, California, United States of America; PLOS: Public Library of Science, UNITED STATES

From pandemics to war, from climate change to disasters–in humanitarian settings, children bear a disproportionate burden. Half of all deaths among children under five years occur in such settings [1]. Globally, one in six children–approximately 450 million–lived in a conflict zone in 2020 [2]. In 2021, children represented 41% of displaced people despite only comprising 30% of the population [3]. Yet, for children, the humanitarian machinery is ill-fit for purpose.

Humanitarian settings often suffer from protracted violence and political and economic instability, which in turn disrupts primary care and public health services such as childhood vaccination and antenatal care. Conflict-affected countries have higher neonatal and child mortality rates, and surviving children are at risk for physical, psychological, developmental, and intergenerational consequences from early trauma [4]. The increasingly protracted nature of conflict and prolonged displacement compound these burdens.

Climate change exacerbates the frequency and intensity of extreme weather-driven disasters and emerging pandemics with catastrophic humanitarian challenges that disproportionately affect children [5].

## A new vision: Humanitarian paediatrics

Despite devastating short- and long-term health and developmental consequences for children affected by crises, the humanitarian system fails to live up to their needs. The global community of professionals working in humanitarian settings lacks consensus on priorities, resource requirements, and training gaps. Our collective response to children’s needs has been inadequate both by dimension and competence.

This discussion aims to elevate the distinct and unmet needs of children and adolescents in humanitarian settings and to propose that these needs can best be met by the purposeful development of a new field: humanitarian paediatrics.

We consider the need for a distinct field of humanitarian paediatrics to be profound. The justification for developing the field is based on the constraints of “global child health” and “humanitarian health.” Global child health is broad and unspecific and, so far, has failed to adequately address the requirements of operating in humanitarian contexts. Similarly, humanitarian health has not kept pace with best paediatric practices. Humanitarian paediatrics can address persistent gaps in the quality and scope of health services for children in humanitarian settings, as well as the lack of child-focused training opportunities and research initiatives [6].

## Children are not small adults

While all people affected by violent conflict or disaster share vulnerabilities and needs, it is essential to recognize the distinct pathophysiological and developmental cadences of childhood. One of the most basic requirements of humanitarian paediatrics is to agree on a common legal, social, or cultural definition of “child” that applies in all settings.

There is a paucity of up-to-date, evidence-based clinical guidelines for treating paediatric patients in humanitarian settings, especially children under five and teenagers; a notable exception is the Neonatal Health in Humanitarian Settings Field Guide [7, 8]. Comprehensive clinical paediatric guidance for healthcare providers in humanitarian settings is needed, as are technical expertise, training, and paediatric-specific medical equipment.

During humanitarian crises, the burden of paediatric trauma and illness can differ substantially from patterns found in settings not characterised by high levels of violence and disruption of essential services. The vulnerability of the individual child is primarily affected by the weakening of familial or communal protective mechanisms [9]. In conflicts, responding to the *direct effects* of combat or displacement on children requires both specialised clinical skills and delivery systems. More distinctive still are patterns of *indirect effects* (due to the destruction of the essentials of life, including food, shelter, health care, etc.). Indirect effects generally reach a scale that far exceeds direct effects, particularly for children and adolescents [4]. Some indirect effects of particular concern are the deterioration of services for perinatal and neonatal health, acute and chronic malnutrition, vaccine-preventable diseases, neglected infectious diseases (including HIV, TB, and measles), and the mental health impacts of violence, forced displacement, and the erosion of community life [10].

There is also an urgent need for clinical protocols that attend to the distinct emotional and psychological needs of children and caregivers experiencing profound trauma (acute, chronic, or repeated). When left untreated, adverse childhood experiences can have lifelong physical, mental and social impacts that shape quality of life, prevent children from achieving their full potential, and impact subsequent generations [10, 11].

## Violence and disruption, the new norm of humanitarian crises

While traditional global child health strategies provide essential guidance, they have not adequately respected the profound implications of extreme violence, disruption of lives, and destroyed infrastructure in generating specific patterns of childhood disease and psychological trauma [10, 11]. Severe material deprivation, the threat of violence, disruption of familial and community protective measures, and the use of food, water, and medical care for coercion or weaponization represent drivers of adverse health outcomes in areas experiencing conflict (9). Humanitarian staff specializing in child health issues must have a fundamental understanding of, and training in, how the political and security dimensions of conflict inform and are ultimately expressed in the epidemiology and clinical conditions encountered in the children and communities they serve [12].

In areas of violent conflict, both patients and health workers continue to be attacked despite explicit international protections for non-combatants and humanitarian workers (9). Schools, early child development centres, and health facilities are deliberately and repeatedly targeted and must be protected [4].

Humanitarian paediatrics must conform to longstanding ethical principles and protocols that depart from more traditional global health and development approaches. It must use a multidisciplinary and de-colonial lens [13]. Principles of humanity, impartiality, neutrality, and independence are paramount, and the Convention of the Rights of the Child provides a protective framework for child health operations [4].

## Paediatric research and training

Paediatric humanitarian research will require the development of designs and analytic methods that are directly relevant to children and sufficiently robust for implementation in insecure environments with weak research and monitoring infrastructure. Research on and with children involves unique ethical concerns as well as practical and protection considerations for which specific training and oversight are essential. Among the most pressing challenges requiring investigation and innovation are the care of premature infants, essential newborn care, intensive neonatal and paediatric care, the prevention and treatment of acute malnutrition and neglected communicable and non-communicable diseases, adolescent-specific care, and mental and psychosocial health [6, 8, 14, 15].

Major humanitarian bodies should integrate humanitarian paediatric training, courses, or focus areas into their learning portfolio. Academic centres engaging with global health should pay particular attention to the specific needs of children in humanitarian contexts. Local actors could highlight the capacity, expertise, and resources existing within a particular context instead of bringing disruptive external standardised support. Given that women and children comprise the majority of beneficiaries of humanitarian action, humanitarian actors must ensure that the workforce has the clinical, ethical, communication, and cultural competencies to provide safe and effective care for them.

We propose to develop a coherent, professional, robust, and global humanitarian paediatrics capacity. This will require a clear delineation of distinct arenas of clinical expertise, research, training, and accountability. We propose that this paediatrics capacity be set on an equal platform that includes communities from crisis-affected countries at the table. Structures set up by the International Paediatric Association could help host such a body.

A broad definition of paediatrics encompasses all health conditions that affect children and incorporates the social, political, and economic dimensions of these conditions. Likewise, humanitarian paediatrics must contribute to a broader coalition of paediatric and humanitarian disciplines. Essential to this is a dual commitment to establishing the elements of humanitarian paediatrics while at the same time strengthening the wider capacity to protect and treat all communities plagued by outbreaks and pandemics, extreme-weather-driven disasters, conflict and war, displacement, and political instability.
